# Use of Near-Infrared Fluorescence Techniques in Minimally Invasive Surgery for Colorectal Liver Metastases

**DOI:** 10.3390/jcm12175536

**Published:** 2023-08-25

**Authors:** Ishaan Patel, Saad Rehman, Siobhan McKay, David Bartlett, Darius Mirza

**Affiliations:** 1 Liver Unit, Queen Elizabeth Hospital, Third Floor Nuffield House, Mindelsohn Way, Birmingham B15 2TH, UK; 2 Royal North Shore Hospital, Reserve Road, St Leonards, Sydney, NSW 2065, Australia; 3 Hon Professor of HPB and Transplant Surgery, University of Birmingham, Edgbaston, Birmingham B15 2TH, UK

**Keywords:** colorectal liver metastases (CRLM), near-infrared fluorescence imaging (NIRF imaging), minimally invasive surgery (MIS), molecular imaging, colorectal cancer (CRC), indocyanine green (ICG)

## Abstract

Colorectal liver metastases (CRLM) afflict a significant proportion of patients with colorectal cancer (CRC), ranging from 25% to 30% of patients throughout the course of the disease. In recent years, there has been a surge of interest in the application of near-infrared fluorescence (NIRF) imaging as an intraoperative imaging technique for liver surgery. The utilisation of NIRF-guided liver surgery, facilitated by the administration of fluorescent dye indocyanine green (ICG), has gained traction in numerous medical institutions worldwide. This innovative approach aims to enhance lesion differentiation and provide valuable guidance for surgical margins. The use of ICG, particularly in minimally invasive surgery, has the potential to improve lesion detection rates, increase the likelihood of achieving R0 resection, and enable anatomically guided resections. However, it is important to acknowledge the limitations of ICG, such as its low specificity. Consequently, there has been a growing demand for the development of tumour-specific fluorescent probes and the advancement of camera systems, which are expected to address these concerns and further refine the accuracy and reliability of intraoperative fluorescence imaging in liver surgery. While NIRF imaging has been extensively studied in patients with CRLM, it is worth noting that a significant proportion of published research has predominantly focused on the detection of hepatocellular carcinoma (HCC). In this study, we present a comprehensive literature review of the existing literature pertaining to intraoperative fluorescence imaging in minimally invasive surgery for CRLM. Moreover, our analysis places specific emphasis on the techniques employed in liver resection using ICG, with a focus on tumour detection in minimal invasive surgery (MIS). Additionally, we delve into recent developments in this field and offer insights into future perspectives for further advancements.

## 1. Introduction

Colorectal cancer (CRC) ranks as the third most prevalent cancer worldwide, and a substantial proportion of patients diagnosed with CRC either present with synchronous or develop metachronous colorectal liver metastases (CRLM) [1,2,3]. Surgical intervention is the primary curative treatment option for CRLM [2]. To facilitate safe and accurate liver transection, intraoperative navigation tools have become necessary, particularly during minimally invasive surgery (MIS). Preoperative planning involves multidisciplinary team (MDT) discussions and the utilisation of preoperative imaging modalities such as computed tomography (CT), magnetic resonance imaging (MRI), or positron emission tomography and computed tomography (PET-CT). Once deemed suitable for resection, patients undergo either open or minimally invasive surgery, sometimes accompanied by radiofrequency ablation (RFA) or microwave ablation (MWA) [3].

In the field of liver surgery, intraoperative fluorescence imaging has emerged as a promising modality for guiding surgical interventions. Near-infrared fluorescence (NIRF) imaging, in particular, has attracted significant interest due to its potential to aid tumour location and demarcation, detecting different patterns of fluorescence in liver lesions, providing guidance for surgical margins, and facilitating anatomically guided resection [4]. The utilisation of the fluorescent dye ICG in NIRF-guided liver surgery has become prevalent worldwide [5] together with the development of enhanced camera systems [4].

Intraoperative ultrasonography (IOUS) is performed to help surgeons during hepatic resection. However, IOUS may miss superficial and subcapsular lesions as well as those smaller than 10 mm [6,7]. Intraoperative imaging modalities, such as NIRF, have shown promise in enhancing surgical precision. NIRF imaging complements IOUS by detecting small capsular and subcapsular lesions that are easily missed by traditional ultrasound [7,8].

The use of NIRF during liver surgery has emerged as an effective imaging technique, [9] offering superior spatial and temporal resolution compared to ultrasound. It employs ICG as the most widely used and studied fluorescent dye, which can be visualised using optimised surgical camera systems. The integration of ICG and NIRF imaging has generated real-time overlay images during both open and minimally invasive surgeries, providing surgeons with valuable feedback [10].

The purpose of this review is to evaluate the efficacy and clinical utility of intraoperative fluorescence imaging, specifically ICG fluorescence, in liver surgery. By synthesising the available evidence from the selected research articles, this review aims to provide an objective and informative assessment of the use of intraoperative fluorescence imaging for tumour detection, anatomical visualisation, and guidance during liver surgery. The review also aims to identify areas of improvement, challenges, and future directions for research and clinical practice in this field.

## 2. Methods

A comprehensive search was conducted in the PubMed database to identify relevant studies for this literature review. The search strategy employed the following keywords: (Near Infrared Florescence) OR (ICG)) OR (Indocyanine Green) AND (Minimal Invasive Surgery) OR (Laparoscopy) OR (Minimal Access Surgery)) OR (Robotic) AND (Colorectal Liver Metastasis)) OR (CRLM).

To ensure a comprehensive and up-to-date review, the search was also performed in the Covidence database. Inclusion criteria were defined as studies that utilised intraoperative NIRF imaging, focused on minimal invasive liver resection (either planned or performed), and were conducted on human subjects. Only English language manuscripts with full-text availability were considered.

The scope of the review encompassed studies that explored tumour detection in the context of CRLM and liver segmentation. Comparative studies, case reports, and case studies that were relevant to the topic were included. Exclusion criteria included studies that involved malignancies other than CRLM, review articles, studies with fewer than five patients, and studies without full-text availability. Conference abstracts, editorials, expert opinions, and review papers were also excluded.

While the number of patients included and the various surgical approaches in the studies were not designated as exclusion criteria, articles were considered relevant and valuable for the completeness of the review.

The primary outcomes of interest in the selected studies were sensitivity, specificity, R0/R1 resection rates, techniques of resection, and the optimal dose and timing of fluorescent dye injection. These outcome measures were deemed essential for evaluating the efficacy and practicality of NIRF-guided surgery in the context of CRLM resection.

## 3. Results

A comprehensive search initially identified a total of 1898 studies published between 1 January 2005 and 30 May 2023. After excluding studies without full-text availability, as well as those not involving human study subjects and those not written in English, 88 studies remained for further evaluation of eligibility based on title and abstract; case series with less than five patients with CRLM were excluded.

The screening process, based on titles and abstracts, led to the exclusion of additional articles. The primary reasons for exclusion were studies conducted on non-human subjects or studies focusing on malignancies other than CRLM.

Subsequently, 54 full manuscripts were reviewed and, after further exclusions, 14 articles were included in the review. 

## 4. Discussion

### 4.1. Basic Properties of ICG

Fluorescence imaging involves the transmission of photons with distinct wavelengths to activate a contrast agent or fluorophore, such as ICG [11]. In surgical applications, these photons navigate through tissue to interact with the fluorophore in the target area. During this process, photons may be absorbed, reflected, or refracted [11]. Absorption by a fluorophore elevates its energy, causing it to enter an excited state before returning to the ground state and emitting a photon. As emitted photons travel through tissues, they encounter reflection, refraction, and scattering, which pose challenges when interpreting and quantifying NIRF images [12].

### 4.2. Mechanism of ICG

The clinical applications of ICG initially focused on liver function and cardiac output evaluation [13]. In the 1970s, a protein-bound form of ICG was discovered to emit fluorescence at around 840 nm when exposed to near-infrared light (750–810 nm) [14]. In addition to its use in liver surgery, ICG has found increasing application in visceral surgeries such as colorectal and upper gastrointestinal procedures, enabling the detection and estimation of vascular perfusion in surgical anastomosis and the visualisation of lymph nodes [13].

Physiologically, ICG is a sterile, anionic, and water-soluble but relatively hydrophobic tricarbocyanine molecule with a molecular mass of 776 Daltons [15]. Upon intravenous injection, ICG rapidly binds to plasma proteins and is swiftly extracted by the liver without undergoing modifications. It is primarily excreted by the liver and appears unconjugated in the bile approximately 8 min post-injection, influenced by liver vascularisation and function [16,17]. The fluorescence of ICG is activated by near-infrared (NIR) light, and the resulting fluorescence can be detected using specialised scopes and cameras [15].

The applications of ICG in liver surgery have garnered significant attention in recent years, encompassing intraoperative guidance for anatomical liver resections, the localization of subcapsular and intraparenchymal tumours, and the identification of biliary anatomy [15]. The unique fluorescence characteristics of ICG, which is metabolised by the liver and excreted through the bile ducts, make it a valuable real-time navigation system for liver surgeons [18]. The dye is accumulated in the liver parenchyma and progressively washed out in normal tissue; meanwhile, it is retained by tumours due to the altered anatomy causing the distortion of peripheral bile structures. Ishizawa et al. previously reported that tumour fluorescence patterns and accumulation timing vary depending on the histological differentiation of the tumours [16,17].

### 4.3. ICG in Liver

ICG is commonly used as a fluorescent dye in hepatic surgery. It exhibits amphiphilic properties and can bind to hydrophilic or lipophilic molecules. ICG actively transfers from plasma to hepatic parenchymal cells and is exclusively excreted through the biliary system [19]. Protein-bound ICG emits light with a peak wavelength of approximately 832 nm when illuminated by near-infrared light [20]. It is preferred to administer ICG using water as a solvent for optimal fluorescent intensity, as saline can promote aggregation [10].

In liver function assessment, the ICG retention test (ICG R15) is a well-known parameter. Impaired clearance is indicated when more than 15% of the injected ICG remains in the plasma after 15 min [21]. The fluorescence properties of ICG in hepatic surgery initially received little attention until Japanese groups started utilizing intraoperative fluorescence cholangiography, which highlighted the biliary excretion properties of ICG [22]. The observation of a specific enhancement pattern of fluorescent light after preoperative ICG injection in patients with hepatocellular carcinoma (HCC) during liver surgery led to increased research on the application of ICG in this field [11].

In anatomic liver resections, the clear identification of liver segment borders is crucial. While IOUS can map major hepatic veins to define hepatic segments, it is insufficient for guiding anatomic resections due to factors like the irregular 3D shape of liver segments, previous chemotherapy, and redo liver resections [23,24]. ICG fluorescence has emerged as a valuable navigation tool in liver surgery, enabling the real-time 3D identification of liver neoplasms and segmental boundaries [19,25,26,27].

Numerous studies have demonstrated the effectiveness of ICG in tumour detection, particularly for identifying previously unknown lesions on liver surfaces or in surgical specimens [11].

### 4.4. Types of Cameras Used

In the field of ICG fluorescence-guided surgery, there are leading companies providing NIR cameras for imaging devices. Hamamatsu Photonics Co’s Photodynamic Eye and Mizuho Medical Co Ltd.’s HyperEye Medical System provide black and white contrast for open-liver settings. Stryker Co’s PINPOINT/SPY PHI and Mizuho Medical Co Ltd.’s HyperEye Medical System feature an overlay mode for simultaneous fluorescence and white-light imaging [28]. The PINPOINT system allows the real-time fusion of colour, monochromatic fluorescence, and fusion images. Newer products like Stryker Co’s 1688 AIM and Karl Storz SE & Co. KG’s Image 1S Rubina incorporate overlay technology. Olympus Corp.’s Viscera Elite II and Intuitive Surgical Inc.’s Firefly offer a fusion image mode and quantitative tissue perfusion assessment, potentially useful in liver parenchymal transection and synchronous CRLM operations [29,30].

Further research is necessary to evaluate the precise surgical margin, liver parenchymal perfusion, and lymph node metastases using the overlay mode. In anatomic liver resections, the overlay mode with high-resolution imaging is the preferred function as it enables the identification of intersegmental and sectional planes, even in deeper liver parenchyma [31,32]. Laparoscopic liver resection using ICG fluorescence imaging has shown utility in achieving negative surgical margins, which may lead to improved surgical outcomes in terms of recurrence and long-term survival. However, more studies and long-term follow-up are required to further investigate this [28].

### 4.5. Clinical Applications

Minimally invasive surgery (MIS) for liver resections poses challenges due to the lack of tactile sensation, difficult location of tumours, absence of 3D vision, and difficulty in performing intraoperative laparoscopic ultrasound, particularly in the posterior region of the liver [29]. Visualising anatomical planes during intraparenchymal transection, especially in cirrhotic patients, redo liver resections, post chemotherapy, and difficult locations of tumours in liver segments, adds to these challenges [33].

Technical differences exist between laparoscopy and robotics. The robotic ultrasound probe differs from the laparoscopic probe, particularly in terms of available probes with an integrated hole for needle guidance in positive staining cases [26,34]. Robotic arms occupy a significant portion of the operative field, making percutaneous needle insertion more challenging. Additionally, in a robotic setting, the operative surgeon is physically distant from the surgical field, necessitating the involvement of another surgeon with expertise in intraoperative ultrasounds and percutaneous needle insertion for positive staining techniques [33].

ICG is utilised to overcome certain limitations in robotic-assisted liver surgery (RALS). Moreover, it accumulates in or around hepatic masses, and integrated near-infrared cameras aid in visualising this accumulation. The influence of ICG staining on surgical and oncological outcomes in RALS patients has been investigated. Multi-centre international studies have evaluated the use of ICG in robot-assisted liver resections [17]. Near-infrared fluorescent (NIRF) imaging with ICG has been studied for various applications, including real-time surgical margin assessment in laparoscopic and robot-assisted resections of colorectal liver metastases. ICG fluorescence imaging has also been proposed as a valuable tool for identifying liver tumours, delineating liver parenchymal territory, and performing cholangiography in open-liver surgery [13,17,33].

Further studies are necessary to explore the benefits in terms of oncological results and the technical feasibility of positive and negative staining techniques, as well as to establish standardised protocols [33].

### 4.6. Staining Pattern

Different staining patterns of ICG have been observed in tumours and their penetration through liver tissue [17]. Hepatocellular carcinoma typically exhibits a total or partial fluorescence staining pattern, while cholangiocarcinoma does not show a predominant pattern [35]. The rim fluorescence staining pattern is commonly observed in CRLM. Wang et al. state that the visualisation of ICG fluorescence may vary among different imaging systems, which could potentially introduce bias [26].

In CRLM, the typical enhancement pattern of ICG after a preoperative IV dose is a rim-shaped pattern [36]. ICG accumulates in the tumour periphery due to immature hepatocytes that cannot excrete it into the bile. Conversely, normal liver parenchyma excretes ICG into the biliary tract within a certain timeframe, resulting in the characteristic enhancement pattern. It is important to consider the limitations of fluorescent light penetration, which is approximately 5–8 mm in liver tissue [37]. Consequently, lesions located deeper than 8–13 mm below the liver capsule may not be directly visualised using near-infrared fluorescence (NIRF) imaging. However, these lesions are still surrounded by ICG, enabling the use of NIRF imaging for assessing resection margins in deeper liver parenchyma, such as during segmental or hemi-hepatectomies [11].

### 4.7. Dose and Timing

The heterogeneity of study designs makes it challenging to establish a consensus regarding the optimal dose of ICG in liver surgery. Various factors, such as the degree of cirrhosis, type of neoplasm (specifically focusing on colorectal liver metastases in this study), patient age, and tumour-associated factors, can affect the visibility of ICG fluorescent emission during surgery. This heterogeneity complicates the creation of a consensus statement on the use of ICG in liver surgery [11].

The dosage and timing of ICG administration vary based on the disease and type of liver parenchyma. Typically, a dose of 0.3–0.5 mg/kg is administered 1–14 days before surgery for normal liver parenchyma [38]. However, in cirrhotic or fibrotic livers with impaired washout properties, the dose should be decreased and should not exceed 0.3 mg/kg. Kobayashi et al. suggested that an additional injection of 2.5 mg of ICG one day before surgery does not increase the false positive rate in cases where there is no ICG retention test or where the interval between injections exceeds 14 days [25].

In cirrhotic or fibrotic livers with impaired liver function, slower metabolic elimination of ICG is associated with an increased false positive rate in tumour detection. For patients with cirrhosis, ICG injection should be performed no less than 4–5 days before surgery [33].

In patients with CRLM who are initially considered to have an unresectable disease, neoadjuvant chemotherapy may be administered to reduce the size and number of CRLM before surgery [36]. The timing of ICG injection in relation to neoadjuvant chemotherapy is still under debate, and no consensus has been reached regarding whether these patients should receive a different dose at a different time point [11].

### 4.8. Applications of ICG Fluorescence Imaging during Liver Surgery

#### 4.8.1. Tumour Location

ICG fluorescence imaging has various applications in liver surgery, including tumour localization. NIRF-guided surgery has demonstrated high sensitivities (ranging from 73% to 100%) in the intraoperative detection of CRLM [17,36,37]. The primary use of ICG fluorescence is in visualising liver lesions, even allowing the detection of neoplasms that may not be identified using IOUS. While IOUS tends to miss superficially located tumours, ICG fluorescence imaging has shown benefits in identifying additional lesions on the superficial surface, up to a depth of 13 mm, that are missed when using IOUS. However, the value of ICG fluorescence in detecting deeper tumours seems to be limited to achieving a proper tumour margin in close proximity to the tumour [11]. There are variances in the R0 rates in different studies; in our opinion, some of these may have very well been due to accepting an R1 resection at the vessel margin due to the location of the tumour of CRLM, bearing in mind the fact that there is no strong evidence to support anatomical resection. However, most studies fail to mention the cases in which R1 resection was accepted at the time of the operation.

The studies that focus on minimal invasive surgery in CRLM are mentioned in Table 1 below.

#### 4.8.2. Anatomical Demarcation

Anatomical liver resections have shown improved oncological outcomes and reduced local recurrence compared to non-anatomical resections, particularly in HCC (39,40). Although these techniques are primarily used for HCC, they can also be applied to CRLM [39,40].

In laparoscopic anatomical liver resections (LALRs), achieving selective inflow portal clamping and portal staining presents challenges, especially for cirrhotic livers and posterosuperior segments. To enhance the precision of anatomical resections and guide intraparenchymal transection, the use of ICG has been incorporated into LALRs [10,20]. Two techniques, positive staining and negative staining, have been employed.

In the positive staining technique, ICG is directly injected into the portal branches responsible for the resected or surrounding territories under ultrasound guidance. This technique allows the tumour-bearing segment to appear green while the remaining liver appears dark under near-infrared light [10,12]. However, the direct injection of ICG into the portal branches requires expertise in laparoscopic ultrasounds and needle punctures [11].

The negative staining technique is more commonly used in laparoscopy, providing a clear demarcation of portal territories. The Glissonian pedicle is isolated using the Glissonian or transfissural approach, and ICG is administered intravenously. The entire liver appears green, while the portal territory to be resected appears dark under near-infrared light [12,19,20]. Negative staining is favoured for hemihepatectomy, sectionectomy, and anterolateral segmentectomy [11].

In the negative contrast technique, ICG administration is utilised to enhance fluorescence in the entire liver except for CRLM. However, the non-specific nature of ICG fluorescence results in a significant false-positive rate (2–20%) in detecting additional lesions, particularly in patients with impaired liver function and a shorter time interval between ICG administration and surgery. The negative staining can fail in the presence of an accessory left hepatic artery, inferior phrenic artery, or other aberrant circulations in the undesired segments [32]. False-positive findings include cysts, hemangiomas, focal steatosis, regenerative nodules, and bile duct proliferation [29,40,41]. Additionally, NIRF imaging allows for the real-time prediction of surgical margins without the need for delayed pathological analysis, which is crucial in minimising R1 resections and improving patient outcomes. NIRF with ICG offers the potential to identify tumour-negative resections, even in cases where achieving an oncologically complete resection with a 1 mm margin may be technically challenging [42].

Both techniques ensure precise anatomical resections, allowing for the identification of intersegmental planes and borders of the resection in both surface and deep liver areas. Proper dissolution and dilution of ICG powder with first sterile water and then with isotonic saline for injection are used to prevent hemolysis [25].

Comparing the two techniques, negative staining has shown higher demarcation, better fluorescence image quality, and greater surgeon comfort and satisfaction in robotic-assisted liver resections [25,29]. However, some experts argue about the priority of positive staining in right superior segments [26].

Further studies are needed to investigate the feasibility and outcomes of these techniques in well-controlled trials, including proper ICG dosing for anatomical liver resections.

#### 4.8.3. ICG in Difficult Locations

Tumours located deep in the right posterior lobe of the liver present challenges for laparoscopic surgery [43]. Funamizu et al. demonstrated that laparoscopic mono-sementectomy using IGG-negative staining showed similar outcomes to traditional techniques for resecting segments 7 and 8 [43]. Li et al. adjusted the surgical position based on tumour location in segments 6 or 7 [44]. ICG use has been explored for centrally located liver tumours to achieve a central hepatectomy [5]. Limited case reports indicate that ICG with negative staining can reduce ischemia in the remaining liver tissue [45,46].

#### 4.8.4. Limitations for Anatomical Resections

The difficulty in re-evaluating segmental boundaries after ICG injection is a major drawback, which can be caused by retrograde flow, unequal blood flow, or the incorrect puncturing of branches or hepatic venous tributaries [47]. Positive staining has limitations in performing on small-diameter branches and carries the risk of reflux, injury to biliary or arterial branches, and outflow of ICG from hepatic veins [48]. Negative staining using the Glissonian approach can be challenging, prone to haemorrhage and biliary lesions, and susceptible to contamination from collateral circulation or multiple portal branch supplies [32]. To prevent contamination, precise investigations of the portal tree and clamping of specific Glissonian pedicles are recommended [32].

Use of ICG in other conditions:

The use of ICG extends to other conditions as well, such as a hybrid approach with interventional radiology involving the preoperative intra-arterial infusion of ICG [31]. Ueno et al. reported on the hybrid approach performed in a hybrid operating room, where preoperative work-up included a 3D reconstruction of portal vein branches to identify anatomical variations and determine the precise injection point and estimated volume to be resected [31]. Moreover, they described a novel positive contrast technique using ICG that was dissolved in an embolic solution and injected into the desired segmental artery, followed by embolization, enabling selective segmental staining and guiding segment resection [31]. However, the technique is currently limited to single-centre/single-intervention cases or prospective cohorts, lacking standardisation in indications, techniques, and results, necessitating further research and prospective studies [31,32].

The intra-arterial ICG hybrid administration technique shows promise but is still in the early stages of development, with use in a limited number of centres [31,49]. 

The challenges in approaching positive or negative staining are complex, requiring perfect use of intraoperative ultrasound for the precise detection of segmental portal branches and skills in percutaneous ultrasound-guided injection with thin needles that accommodate position variations due to respiratory movements and cardiac activity [32]. Teamwork is also essential for ICG dilution and preparation, as well as for the synchronisation of clamping and intravenous injection in the case of negative staining [32].

Liberale et al. demonstrated the equivocal role of ICG fluorescence in the detection of peritoneal metastases due to colorectal cancer [38]. Especially, mucinous adenocarcinoma showed a specific fluorescence distribution, as indicated by a hyperfluorescent peripheral rim and hyperfluorescent centre, in contrast to non-mucinous adenocarcinoma, which ubiquitously expressed hyperfluorescent emission [50].

#### 4.8.5. Use of ICG in Bile Duct Obstruction and Preoperative PV Embolisation

ICG has been investigated for its potential use in bile duct obstruction and preoperative portal vein embolization (PVE). It has been shown to aid in identifying cholestatic regions affected by bile duct obstruction and altered biliary excretion, which can help spare healthy liver tissue during tumour resection [51,52]. In the case of preoperative PVE, ICG systemic injection can facilitate the visualisation of the transection plane without liver manipulation, serving as a negative staining method [53,54].

### 4.9. Recent Developments and Future Perspectives

#### 4.9.1. Real-Time Imaging

The surgical technique in CRLM uses the fluorescent rim after ICG injection as the resection margin [55]. Early results show promise in achieving tumour-negative resections by removing the entire fluorescent rim for R0 resection. However, further validation is needed, and certain patients with local recurrence or scar tissue may have atypical tumour rims. The study by Achterberg et al. suggests that real-time ICG fluorescence-guided surgery has the potential to improve tumour margins in minimally invasive liver metastasectomies, and ongoing MIMIC trial will provide more insights regarding this [55].

In the prospective multicenter MIMIC trial, the accuracy of in vivo ICG fluorescence-guided liver surgery is being evaluated. The aim is to investigate whether the use of NIRFimaging during minimally invasive resection of CRLM can predict the tumourmargin in vivo, which may lead to higher radical resection rates [11]. Consecutively, long-term, disease-free survival and overall survival will be studied. The negative predictive value (absence of fluorescence in the resection plane) of NIRF was 92% for an R0 resection. In 21% of CRLM resections, a change in surgical management was documented based on ICG fluorescence [11]. In patients undergoing minimally invasive CRLM resections, the absence of ICG fluorescence predicts a tumour-negative margin with high accuracy and leads to a change in surgical strategy in more than one-fifth of patients [11].

#### 4.9.2. Tumour-Specific Imaging Probes

Fluorescence intensity in tumour identification is commonly quantified using the signal-to-background ratio (SBR) or tumour-to-background ratio (TBR), with a TBR of 1.5 or higher considered sufficient. Several tumour-targeted fluorescent agents, such as SGM-101, cRGD-ZW800-1, bevacizumab-800CW, and ONM-100, have shown promise in early clinical studies for colorectal cancer (CRC) [56]. SGM-101, specifically, has demonstrated successful visualisation of malignant lesions in CRC patients and is being studied as a tumour-specific fluorescence imaging probe for CRLM [9,55,57]. Further research is needed to compare the effectiveness of SGM-101 with ICG and assess its potential benefits in patients with primary tumours, lymph node metastases, or peritoneal metastases expressing carcinoembryonic antigen (CEA). Using binding moieties, such as antibodies targeting CEA, can help improve the specificity of NIRF imaging and reduce false-positive rates in CRLM and CRC [49]. Additional investigations are exploring alternative targets for imaging, expanding the possibilities for CEA-negative tumours [57].

#### 4.9.3. Use of AI with ICG

In a study by Hardy et al., the application of Artificial Intelligence/Machine Learning techniques demonstrated the ability to identify CRLM and map their fluorescence perfusion patterns using 2D mapping [58]. This approach may have implications for reducing positive margin rates during metastasectomy and detecting additional metastases. The study involved a cohort of 24 patients, and the Machine Learning algorithm achieved a correct classification rate of 97.2% for CRLM and all benign lesions within 90 s of ICG administration. The algorithm utilised mathematical curve analysis to identify ICG inflow/outflow differentials between healthy liver tissue and CRLM, providing valuable insights for improved surgical decision-making [57].

## 5. Conclusions

In conclusion, ICG fluorescence imaging has shown potential in various aspects of CRLM surgery. It offers benefits in tumour localization, anatomical resections, and the identification of tumour-negative margins; however, more studies are required in future to understand its full impact. Tumour-specific imaging probes, including SGM-101, hold promise for improving specificity and targeting CEA-negative tumours. Further research is needed to optimise ICG dosing, standardise techniques, and investigate the feasibility and outcomes of ICG-guided surgery in well-controlled trials (Supplementary Figure S1).

## Figures and Tables

**Table 1:** Showing studies focusing on tumour demarcation for CRLM in minimal invasive surgery.

Author	Year	Country	Design	No.of Patients (Lesions)	Lap/Open/Robotic	Dose and Timing	FP (%)	Sensitivity of NIR for Detecting CLM (%)	No.of Additional Lesions	R0 (%)	Tumour Type
Marino et al.	2020	Spain	Prospective cohort	40 (52)	Robotic	0.5 mg/kg 5 days	40	88	yes	100	HCC/CLM
Aoki et al.	2019	Japan	Prospective cohort	14 (nad)	Lap	nad	nad	nad	nad	nad	HCC/CLM
Aoki et al.	2018	Japan	Prospective cohort	25 (30)	Lap	0.5 mg/kg 2–14 days	nad	nad	yes	100	HCC/CLM
Terasawa et al.	2017	Japan	Prospective cohort	41 (53)	Lap	0.5 mg/kg 1–3 days	nad	85	yes	nad	CLM
Lim et al.	2021	Singapore	Prospective cohort	32 (46)	Lap/Open	0.25 mg/kg 4–7 days	nad	100	nad	88	HCC/CLM
Boogerd et al.	2017	The Netherlands	Prospective cohort	22 (46)	Lap	10 mg, 1 day	nad	92	yes		HCC/CLM
Handgraaf et al.	2017	The Netherlands	Retrospective cohort	86 (nad)	Lap/Open	10–20 mg, 1–2 days	nad	100	yes	nad	CLM
Kudo et al.	2014	Japan	Prospective cohort	17 (32)	Lap	0.5 mg/kg within 14 days	nad	69	yes	nad	HCC/CLM
Takahashi et al.	2016	USA	Prospective cohort	15 (62)	Lap/Open/Robotic	7.5 mg 1–2 days	nad	53 (100 for superficial tumours)	yes	nad	CLM
Patel et al.	2022	UK	Prospective cohort	15 (30)	Lap/Open	10 mg, 1 day	nad	93	yes	71	CRLM
Mehdorn et al.	2021	Germany	Prospective cohort	20 (nad)	Robotic	25 mg in 50 mL, 1 day	nad	nad	yes	85	HCC, CRLM, Other liver mets
Kunshan et al.	2022	China	Retrospective cohort	32 (73)	Lap/Open	0.25 mg/kg 1 day	8	nad	yes	nad	CRLM
Achterberg et al.	2020	The Netherlands	Prospective cohort	16 (16)	Lap/Robotic	10 mg, 1 day	nad	nad	yes	50	CRLM
Weixler et al.	2023	Germany	Prospective cohort	66 (66)	Open/Lap	10 mg, 1 day	21	nad	yes	64	CRLM, HCC, Other met

Note: nad = No available data.

## Data Availability

No new data were created or analyzed in this study. Data sharing is not applicable to this article.

## Supplementary Materials

The following supporting information can be downloaded at: https://www.mdpi.com/article/10.3390/jcm12175536/s1, Figure S1: ICG-positive colorectal cancer liver metastasis showing fluorescent rim. (A) Color image of colorectal cancer liver metastasis; (B) near-infrared light image of colorectal cancer liver metastasis; (C) merged image of colorectal cancer liver metastasis.
