# Supplementary material for: Use of Near-Infrared Fluorescence Techniques in Minimally Invasive Surgery for Colorectal Liver Metastases
Source: J Clin Med. 2023 Aug 25;12(17):5536. doi: 10.3390/jcm12175536 (PMC10488819; doi:10.3390/jcm12175536)
Supplement: Supplementary file 1 [file jcm-12-05536-s001.zip › jcm-2537625-supplementary.pdf]

Supplementary Figure S1.

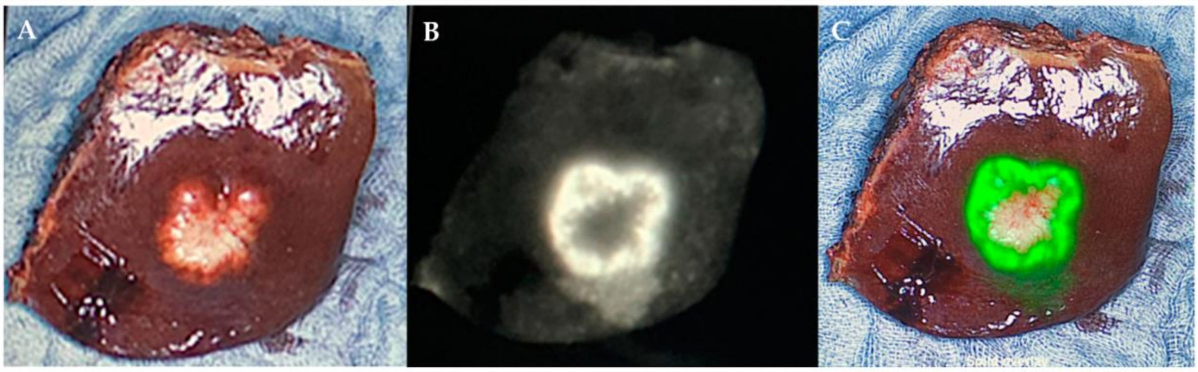


Figure S1 ICG-positive colorectal cancer liver metastasis showing fluorescent rim. (A) Color image of colorectal cancer liver metastasis; (B) near-infrared light image of colorectal cancer liver metastasis; (C) merged image of colorectal cancer liver metastasis.
